# CpG oligonucleotides bind TLR9 and RRM-Containing proteins in Atlantic Salmon (*Salmo salar*)

**DOI:** 10.1186/1471-2172-14-12

**Published:** 2013-03-01

**Authors:** Dimitar B Iliev, Ingrid Skjæveland, Jorunn B Jørgensen

**Affiliations:** 1 Norwegian College of Fishery Science, University of Tromsø, N-9037, Tromsø, Norway

**Keywords:** Innate immunity, Toll-like receptors, CpG oligonucleotides, Teleosts, RNA recognition motifs

## Abstract

**Background:**

Bacterial DNA is well-known for its potent immunostimulatory properties which have been attributed to the abundance of CpG dinucleotides within the genomes of prokaryotes. Research has found that mammalian TLR9 is a receptor which mediates the immune response to CpG DNA; however, its functional properties in non-mammalian vertebrates are still poorly characterized. Leukocytes isolated from lower vertebrates, including teleosts, respond to CpG DNA and TLR9 has been identified in many fish species; however, the ligand-binding properties of fish TLR9 have, so far, not been studied. The fact that some vertebrates, like chicken, lack TLR9 and use an alternative molecule (TLR21) as a receptor for CpGs has questioned the functional conservation of TLR9 within vertebrates.

**Results:**

In the current study, TLR9 from Atlantic salmon (SsTLR9) has been found to interact with synthetic oligonucleotides via a CpG-independent but a pH-dependent mechanism. The endogenous receptor, expressed by primary mononuclear phagocytes colocalizes with CpG oligonucleotides (ODNs) in vesicles that appear to be endosomes. When overexpressed in salmonid cell lines, SsTLR9 spontaneously activates ISRE-containing promoters of genes involved in the IFN response; however, the transgenic receptor fails to translocate to CpG-containing endosomes. This indicates that only specific immune cell types have the ability to relocate the receptor to the appropriate cellular compartments where it may become activated by its ligand. In addition, through co-precipitation and mass spectrometry, two salmon proteins - hnRNPA0 and NCOA5, which both contain RNA-binding domains (RRM), were found to bind CpG ODNs, suggesting they may be involved in the CpG response in salmon leukocytes.

**Conclusion:**

The presented data are the first to demonstrate that the DNA-binding properties of TLR9 are conserved between teleosts and mammals. The current study also identifies additional molecules which may function as mediators of the immunostimulatory properties of foreign DNA.

## Background

Bacterial DNA is a potent immunostimulant [1] and research has found that the abundance of CpG dinucleotides (CpGs) in genomes of prokaryotes is a major factor contributing to its immunostimulatory properties [2]. Toll-like receptor 9 (TLR9) has been identified as a major player in the innate immune response to bacterial DNA and synthetic oligodeoxynucleotides (ODNs) that contain unmethylated CpG motifs [3].

The members of the TLR family are well-conserved type I transmembrane proteins characterized by a leucine-rich domain involved in ligand recognition and a toll/interleukin-1 receptor (TIR) intracellular signalling domain [4]. Acting as innate immune receptors, members of this family have been found to mediate the response to different bacteria- and virus-derived molecules, including lipopolysaccharide, bacterial lipopeptides [5], double-stranded RNA [6] and CpGs [3].

Due to the importance of foreign- and host-derived DNA for induction of protective reactions against pathogens and autoimmune disorders, respectively, a great deal of research has been devoted to studying the immunostimulatory properties of CpGs and the function of TLR9 (reviewed in [7]). In mammals, the receptor is found in the endoplasmic reticulum in resting immune cells. Upon exposure of the cells to CpG DNA, TLR9 translocates to the endosomal compartments [8], where it may interact with its ligand. The binding of the receptor to DNA seems to be sequence independent but it requires low pH conditions and proteolytical activation [9]. Once activated, the receptor signals through Myeloid differentiation primary response gene 88 (MyD88) to activate transcription factors including nuclear factor kappa-B (NFkB) and interferon-regulatory factor 7 (IRF7) involved in the upregulation of proinflammatory genes and type I interferon (IFN), respectively [7].

The function of TLR9 is relatively well-characterized in mammals; however, its function in lower vertebrates remains obscure. TLR9 has been identified in teleosts [10-14], and the expression of the protein in Atlantic salmon (*Salmo salar*) leukocytes is inducible by IFN-γ [15]. It has been shown that the bacterial DNA and CpG ODNs are able to activate immune cells in fish [16,17]. In particular, research has found that the upregulation of proinflammatory cytokine in CpG-stimulated trout macrophages relies on efficient endosomal acidification [18]. Like in mammals, salmon leukocytes are able to distinguish between different classes of CpG ODNs by upregulating type I IFN and stimulating lymphocyte proliferation [19] and data suggests that in Japanese flounder (*Paralichthys olivaceus*) TLR9 is involved in the up-regulation of a tumor necrosis factor-alpha (TNFα) promoter by CpGs [11]. These data indicate that the TLR9 function is conserved throughout the vertebrate classes; however, direct interaction between stimulatory CpG ODN and TLR9 has not been demonstrated in non-mammalian vertebrates, so far.

The major goal of the current study has been to characterize the ability of a salmon TLR9 homolog (SsTLR9) to interact and mediate the response to immunostimulatory, phosphorothioate (PS)-modified CpG ODNs. The results demonstrate that the binding of SsTLR9 to PS-modified ODNs is not dependent on the presence of CpGs; however, the affinity of this interaction is pH-dependent. Transgenic SsTLR9 is able to spontaneously activate IFN-stimulated response element (ISRE)-containing promoter constructs. Nevertheless, unlike in primary leukocytes, it fails to colocalize with CpG ODNs when overexpressed in salmonid cell lines. The study further identifies proteins that contain RNA recognition motifs (RRM), including heterogeneous nuclear ribonucleoprotein A0 (hnRNPA0) and nuclear receptor coactivator 5 (NCOA5), as potential mediators of the effects of exogenous DNA in salmon immune cells.

## Results

### SsTLR9 binds phosphorothioate-modified ODNs through a CpG-independent but a pH-dependent mechanism

**Figure 1:**
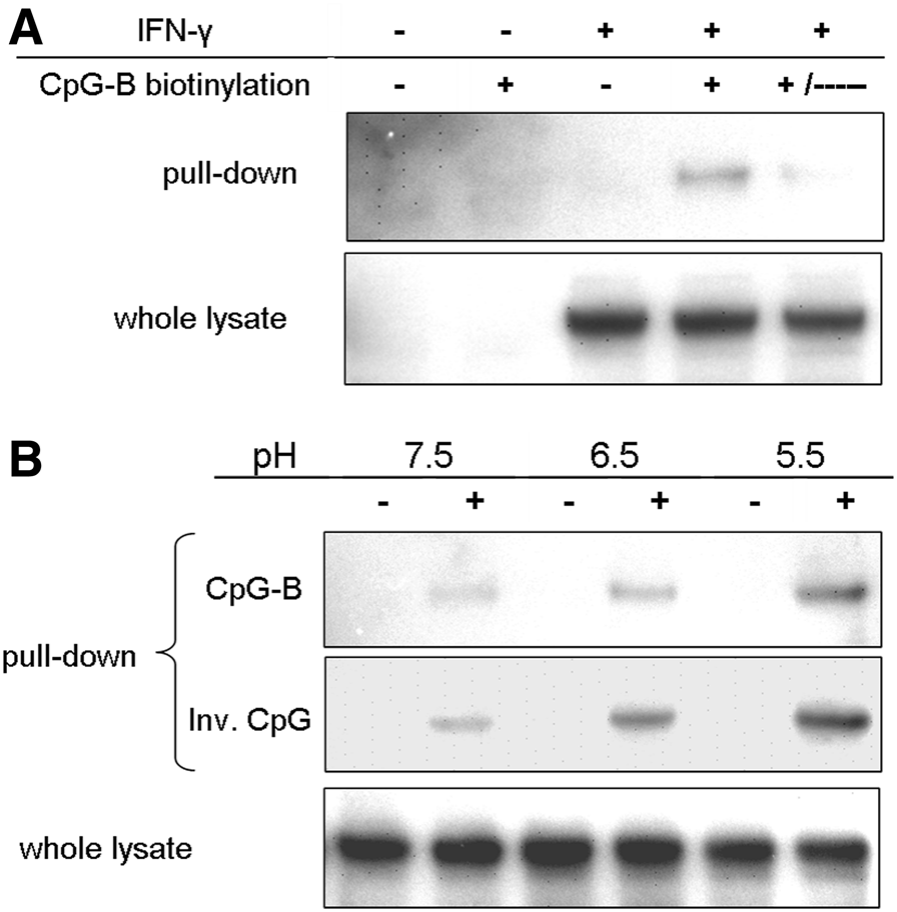
**SsTLR9 binds PS-modified ODNs through a CpG-independent but a pH-dependent mechanism. A**, Specific binding of CpG-B ODNs to SsTLR9. To up-regulate the SsTLR9 protein, primary salmon mononuclear phagocytes were pretreated with IFN-γ for 48 hours, as indicated. The cells were further stimulated with either biotinylated “+” or non-biotinylated “-” CpG-B (2 μM) for 2 hours. To further confirm the specificity of the binding, a sample was treated with biotinylated CpG-B in the presence of 10 μM non-biotinylated CpG-B (indicated with “+/–”). Following the stimulations, cell lysates were subjected to a pull-down analysis with streptavidin-conjugated magnetic beads and then analyzed on WB with SsTLR9 ab. In addition, the whole lysates were analyzed in parallel to confirm the SsTLR9 up-regulation and loading. In the pull-down blot, the SsTLR9 signal (~120 kDa) is detectable only in the samples stimulated with biotinylated CpG ODNs. The addition of an excess (5-fold higher concentration) of non-biotinylated ODNs significantly reduces the amount of the co-precipitated SsTLR9 which further confirms the specificity of the binding of the receptor to the ODNs. **B**, The interaction between ODNs and SsTLR9 is enhanced under low pH conditions. The pull-down was performed under the conditions described above and the pH of the lysis buffer was adjusted to the indicated values using 3 M HCl. In addition, inverted non-stimulatory CpG-B (ODN 2007 T) was included in the experiment. The results demonstrate that SsTLR9 binds to both the CpG and the inverted CpG ODNs with similar avidity which was highest at pH 5.5. The results shown in both panels were confirmed in at least two independent experiments.

A pull-down approach was used in order to find out whether SsTLR9 is able to bind CpG ODNs. Adherent mononuclear phagocytes isolated from head kidney were first stimulated for 48 hours with IFN-γ [20] in order to upregulate the SsTLR9 protein [15] and then stimulated for 2 hours with biotinylated CpG-B prior to lysis and coprecipitation with streptavidin-conjugated magnetic beads. The Western blot (WB) results shown in Figure 1A demonstrate that SsTLR9 coprecipitates with the streptavidin beads only when the cells have been treated with biotinylated but not with non-labelled CpG-B ODNs. Furthermore, addition of a 5-fold higher dose of non-labelled CpG-B (10 μM) to the biotinylated CpGs prior to stimulation significantly reduced the amount of the coprecipitated SsTLR9, which further confirmed the specificity of the binding. TLR9 is known to interact with DNA in acidified organelles including endosomes and lysosomes [8]. To analyse the efficiency of the binding of CpG ODNs to SsTLR9 under different pH conditions, the acidity of the lysis buffer was adjusted using HCl. The results presented in Figure 1B show that the efficiency of coprecipitation of both CpG-B and non-stimulatory, inverted CpG-B ODNs with TLR9 increases gradually with the reduction of the pH from 7.5 to 5.5. Therefore, the binding of SsTLR9 to ODNs is independent of their immunostimulatory capacity and is most efficient under low pH.

### SsTLR9 up-regulated by CpG-B and IFN-γ translocates to endosomal compartments where it colocalizes with its ligand

**Figure 2:**
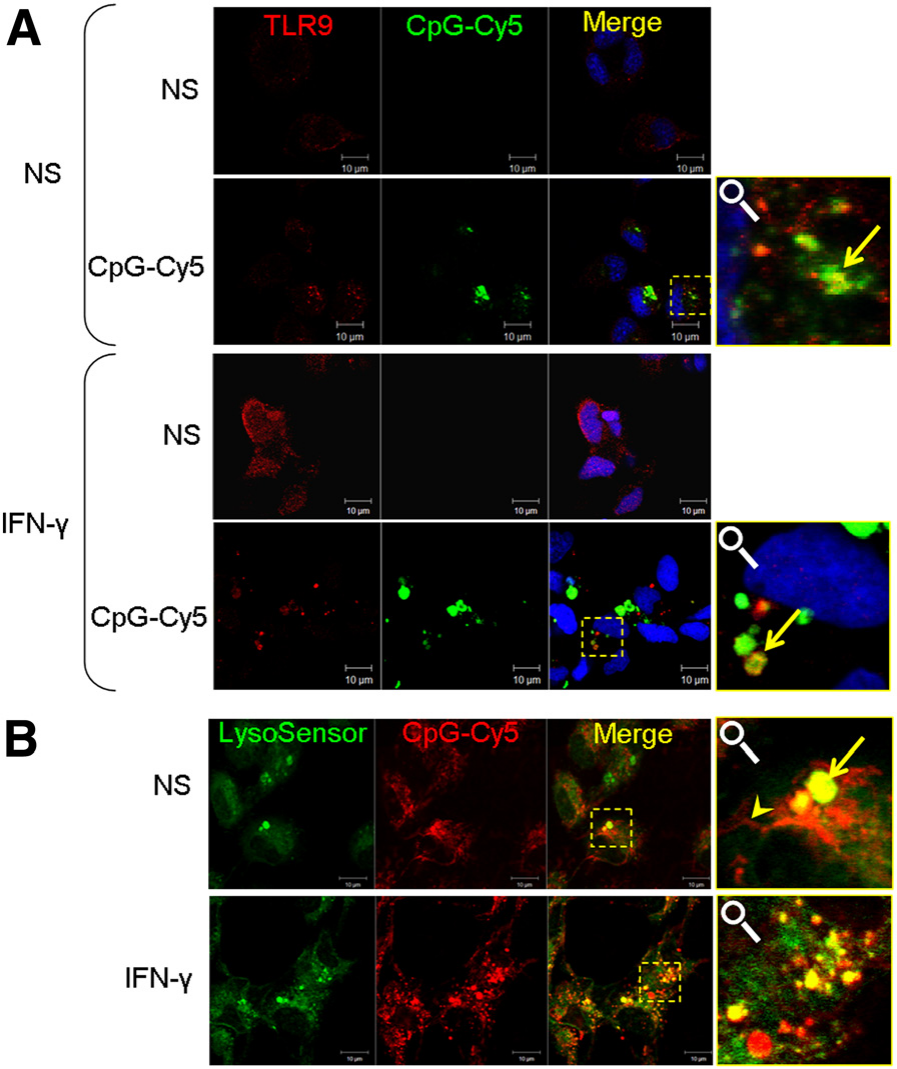
**CpG ODNs colocalize with endogenous SsTLR9 in primary salmon mononuclear phagocytes. A**, The head kidney-derived cells were incubated on coverslips and following the indicated treatments the cells were processed for immunostaining and confocal microscopy as described in “Methods”. Treatment with fluorescent CpG-Cy5 (green pseudocolor) for 24 hours modestly up-regulated SsTLR9 which was detected in small granules. Pretreatment with IFN-γ for 48 hours enhances the SsTLR9 expression which upon addition of CpG-B is transported to vesicles that also contain CpG ODNs. Colocalization visible as yellow coloring in the merged images is indicated with arrows in the magnifications. The nuclei were stained with SYTOX Green (blue pseudocolor). NS stands for “no stimulation”. **B**, In live cells, CpG-B accumulates in tubular structures (arrowhead) and acidified vesicles (arrow) which, most likely, represent maturing endosomes. The cells were stimulated as described for panel A. Endolysosomes were stained with LysoSensor Green (pKa 5.2) and the cells were observed alive.

In order to interact with its ligand and to initiate signalling, TLR9 needs to be translocated from endoplasmic reticulum to the endosomes [8]. In the current study, immunostaining combined with confocal microscopy analysis confirms that both in CpG and IFN-γ-treated mononuclear phagocytes, SsTLR9 colocalizes with fluorescent CpG-B in granules and vesicles (Figure 2A). While SsTLR9 staining in resting phagocytes is not clearly distinguishable from the background, stimulation with CpG-B for 24 hours results in detectable signal, most of which is found in cytoplasmic granules and speckles where it colocalizes with the endocytosed CpGs. The results presented in Figure 2B indicate that in primary mononuclear phagocytes, the endocytosed CpGs accumulate in tubular structures and vesicles. Most of the vesicles are LysoSensor-positive indicating that these are maturing endolysosomes. Priming the cells with IFN-γ results in increased accumulation of CpGs in endolysosomes which correlates with the enhanced SsTLR9 expression and colocalization with CpGs, as compared to non-primed cells (Figure 2A). LysoSensor staining is not compatible with immunocytochemistry since it is destroyed by membrane permeabilization. However, the fact that most of the CpG positive vesicles in IFN-γ primed cells are LysoSensor-positive and that a considerable number of them, are SsTLR9-positive indicates that it is very likely that at least some of the SsTLR9-CpG interaction occurs in endolysosomes.

### SsTLR9, overexpressed in Chinook salmon embryo (CHSE) cells, spontaneously upregulates the transcriptional activity of ISRE-containing promoters but the receptor fails to respond to CpG-stimulation

**Figure 3:**
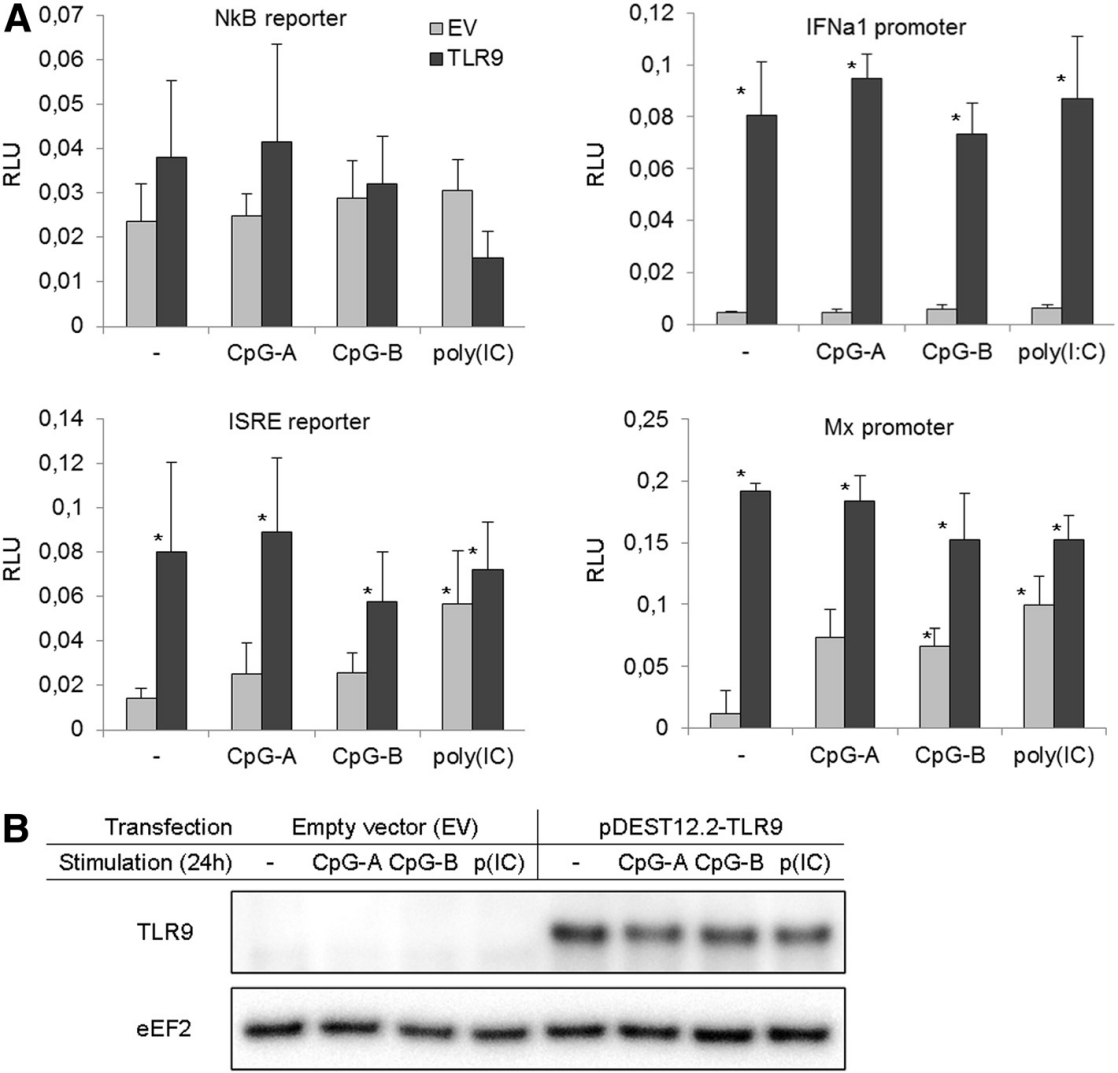
**Transgenic SsTLR9, overexpressed in CHSE cells, spontaneously upregulates ISRE-containing promoters but it does not affect the response to CpGs. A**, The cells were cotransfected with the indicated firefly luciferase reporter constructs and either a SsTLR9-expressing plasmid or the empty vector (EV), as indicated. After incubation for 48 hours, to allow for accumulation of transgenic protein, the cells were stimulated for 24 hours with CpG-A, CpG-B and poly(IC). The inducible expression of the firefly luciferase was normalized using the control Renilla luciferase values and is presented as relative luciferase units (RLU). Overexpression of SsTLR9 upregulated the activity of all of the tested promoters except the NFkB reporter. The stimulation of control cells with CpGs and poly(IC) also activated these promoters with variable intensity. The combination of SsTLR9 overexpression and stimulation gave no enhanced response. The error bars show the standard deviation, N = 5, *P < 0.05 vs. EV controls. **B**, CHSE cells do not express detectable endogenous TLR9 protein. The lysates used for the RGAs were analysed on WB with the SsTLR9 ab. As the antigenic peptide used for the generation of the ab is conserved across *Salmo* and *Oncorhynchus* taxa, the ab is expected to recognize the Chinook salmon homolog with similar affinity.

Reporter gene assays (RGAs) are commonly used to assess the function of genes through a “gain of function” approach. Namely, overexpression of immune molecules such as TLR homologs and their downstream signalling mediators in cells lines that don’t express these proteins may result in an enhanced response to the ligands that require their presence. In the current study, CHSE cells were used for the RGAs as these cells are transfectable (up to 20-30%; Iliev, unpublished observations) with transfection reagents with low toxicity such as FuGene (Roche). As shown in Figure 3A, after transfection with a plasmid that expresses SsTLR9 under the control of the Cytomegalovirus (CMV) promoter, the activity of the salmon IFNa1 promoter, the trout Mx1 promoter and a promoter containing tandem ISRE repeats were significantly upregulated. In all of these promoters ISRE elements are essential for their activity [21,22]. In contrast, the activity of the NFkB promoter construct did not seem to be affected under these experimental conditions. When control (EV-transfected) cells were stimulated with CpG-A, CpG-B and poly(IC), the ISRE and the Mx promoters were modestly activated. The upregulation was most pronounced with the Mx promoter and upon poly(IC) stimulation. No synergistic promoter upregulation could be observed when TLR9-transfected cells were incubated with any of the stimulants.

Aliquots from the RGA samples were analysed by Western blotting to check the expression of the transgenic SsTLR9 and to investigate whether endogenous TLR9 is expressed in CHSE cells. Results shown in Figure 3 demonstrate that, while the expression of the transgenic SsTLR9 is abundant, no sign of endogenous SsTLR9 can be detected in the stimulated samples, even after the WB membrane has been developed with an ultra-sensitive substrate (as described in the “Methods” section). The TLR9 mRNA expression in CHSE cells was analysed with a Taqman assay. The analysis did not detect TLR9 mRNA in non-treated CHSE cells while only weak expression was detected after the cells had been stimulated with IFNa1 for at least 48 h (results not shown).

### CHSE cells accumulate CpGs in late endosomes and lysosomes but fail to translocate ectopically expressed SsTLR9 into these compartments. Also, in the salmon head kidney-derived cell line TO, no colocalization between the CpGs and SsTLR9 was found

**Figure 4:**
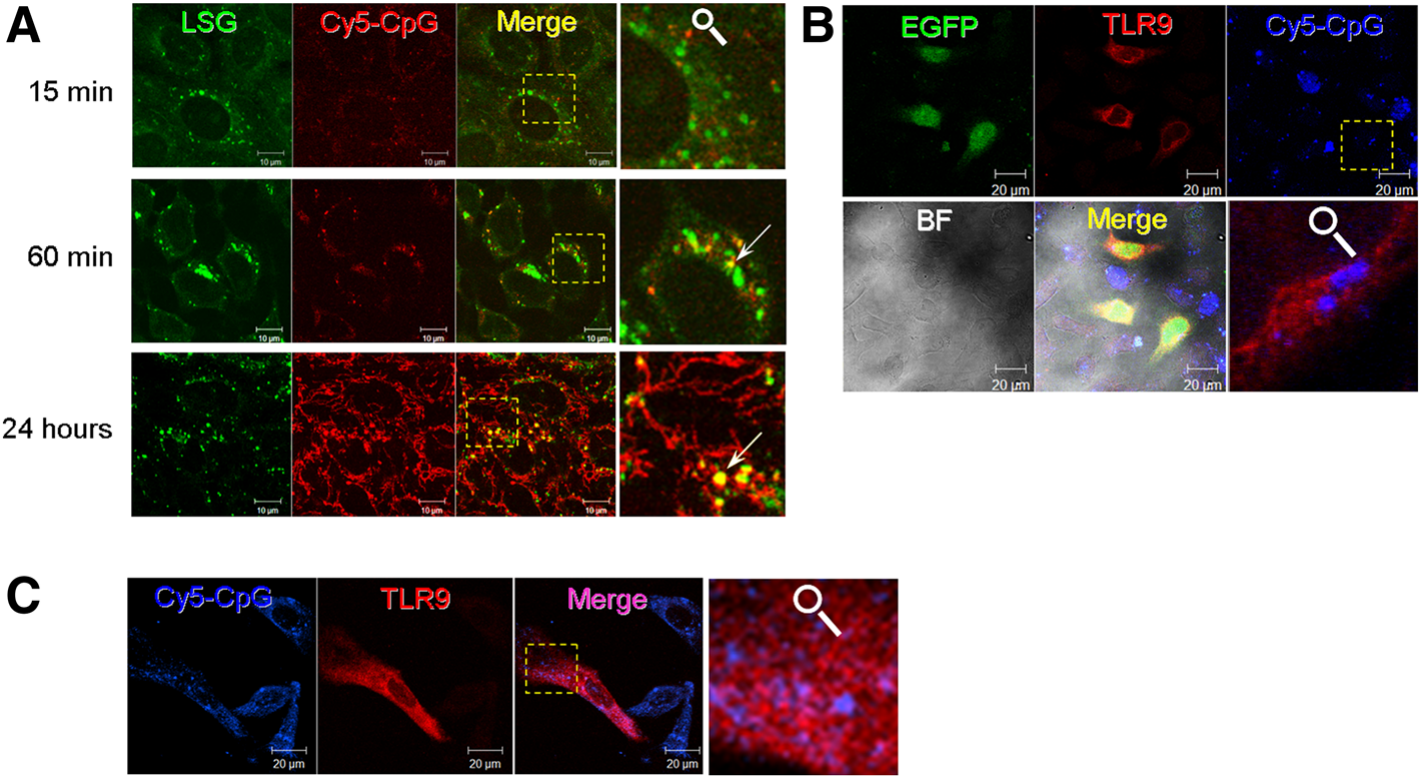
**CHSE cells accumulate significant amounts of CpGs in endolysosomes but fail to relocate transgenic SsTLR9 into these compartments; similarly, TO cells, a salmon head kidney-derived cell line do not translocate CpGs into the CpG-containing compartments. A**, Live CHSE cells at different time points after addition of Cy5-CpGs (red). Late endosomes and lysosomes are stained with LysoSensor Green (LSG). Endocytosis of the CpG ODNs is visible within 15 min in small vesicles without clear colocalization with the LysoSensor-stained endolysosomes. Colocalization with LysoSensor Green is detectable within 60 min indicating transition of the endocytosed CpGs from early to late endosomes and lysosomes. Within 24 hours, significant amounts of CpGs are detected both in acidic vesicles and in tubular structures, the later of which most likely represent tubular endosomes/lysosomes. **B**. Unlike the endogenous SsTLR9 detected in primary cells (Figure 2), SsTLR9 overexpressed in CHSE cells does not colocalize with CpGs. To label transfected cells as an indicator of the specificity of the staining, the plasmid expressing TLR9 was mixed with a plasmid expressing EGFP (green). Cy5-CpGs are shown in blue pseudocolor. **C**, same is observed in salmon TO cells, a poorly-transfectable cell line derived from salmon head kidney. BF stands for bright field. The magnified areas are indicated with dashed boxes.

To test whether CHSE cells can efficiently accumulate CpG ODNs in endolysosomal compartments, the cells were stimulated for different periods with fluorescent CpGs. The late endosomes and lysosomes (pH < 5.5) were stained with LysoSensor Green and the colocalization was analysed with confocal microscopy. Within 15 min of stimulation, CpG ODNs could be detected in small vesicles; however, at this time point, there was no clear colocalization with LysoSensor-positive vesicles (Figure 4A). After 60 min, some of the endocytosed CpGs entered the acidic vesicles indicating transition from early to late endosomes. At 24 hours post-stimulation, significant amounts of CpG-B ODNs were accumulated in late endosomes and lysosomes. In addition, the fluorescent CpGs could be observed in tubular structures which most likely represent the tubular lysosomal system. These data indicate that CHSE cells have the capacity to accumulate large amounts of CpG and to redistribute them from early endosomes to acidified vesicles and tubular compartments of the endo-lysosomal system.

To determine the intracellular distribution of ectopically expressed SsTLR9, CHSE cells were cotransfected with an SsTLR9-expressing plasmid and a vector expressing enhanced green fluorescent protein (EGFP). The EGFP expression was used as a positive control to identify transfected cells. When the transfected CHSE cells were stained with the SsTLR9 antibody, specific signal was observed only in cells that express the cotransfected EGFP confirming the specificity of the immunostaining (Figure 4B). Although these cells had accumulated significant levels of CpG ODNs 24 hours after stimulation, unlike in primary mononuclear phagocytes, no colocalization between transgenic SsTLR9 and Cy5-labelled CpG ODNs could be observed. Similar results were obtained when TO cells, a cell line derived from salmon head kidney was used (Figure 4C).

### Isolation of CpG-B ODN-binding proteins from primary salmon mononuclear phagocytes. Cytoplasmic EGFP-hnRNPA0 fusion protein colocalizes with CpG-B ODNs

**Figure 5:**
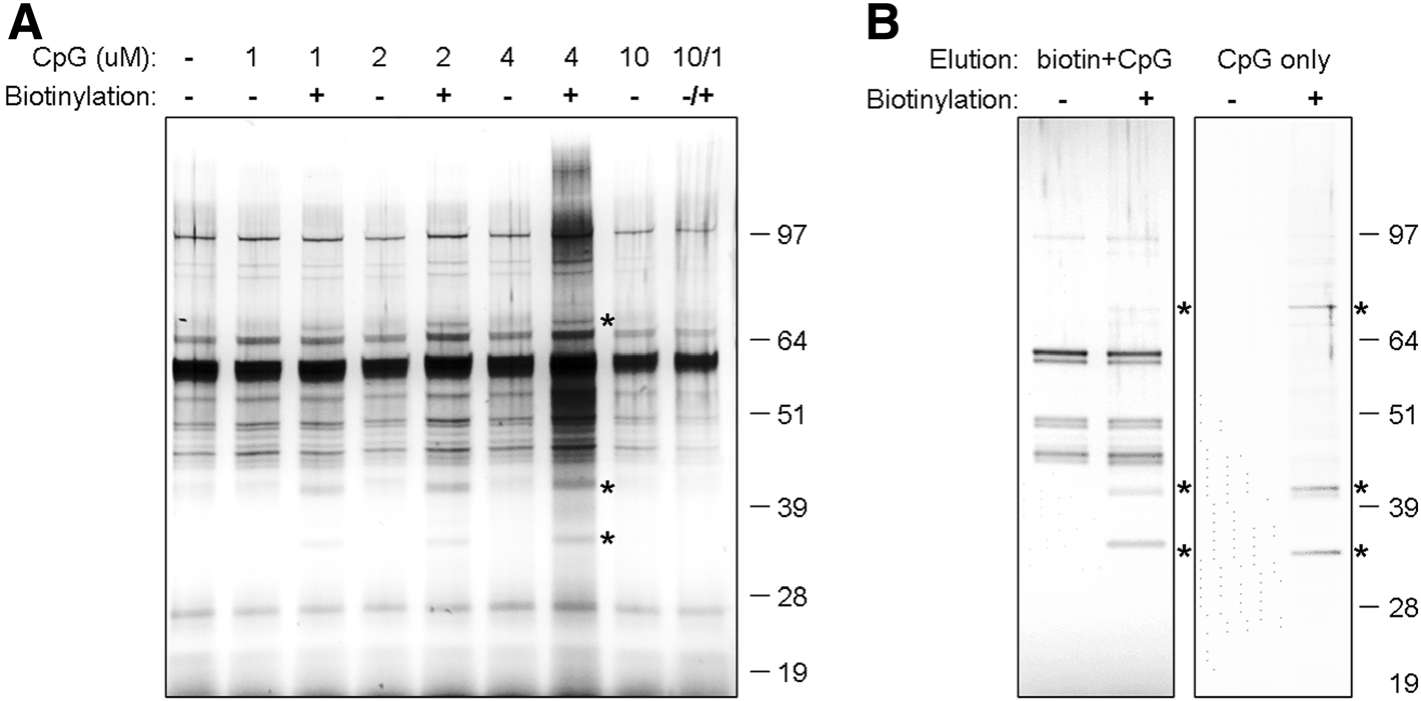
**Isolation of CpG-B ODN-binding proteins from primary salmon mononuclear phagocytes. A**, Image of a silver-stained polyacrylamide gel showing ODN-specific, dose-dependent co-precipitation of putative CpG-B ODN-binding proteins. The cells were incubated with the indicated concentrations of biotin-labeled and/or non-biotinylated CpG-B ODNs prior to co-precipitation as described in Figure 1A. The samples were further boiled in LDS sample buffer and subjected to PAGE and silver staining. Addition of an excess of unlabelled CpG-B inhibits the co-precipitation of the bands indicated with “*” suggesting that these represent proteins that specifically bind to the ODNs. **B**, In order to identify the CpG-binding proteins through MS, milder elution techniques were used either through a combination of biotin and CpGs (left) and CpGs only (right) as described in “Methods”.

The pull-down experiments in the current study demonstrate that SsTLR9 expressed in primary head kidney mononuclear phagocytes specifically coprecipitates with CpG ODNs. To identify other molecules that might be involved in the CpG recognition and signalling, proteins that coprecipitated with biotinylated CpGs were analysed with PAGE/silver staining and mass spectrometry. Initially, the cell isolation, stimulation and isolation was performed as in the experiments used for the characterization of SsTLR9 binding. As a result, in addition to the many non-specific bands visible on the silver-stained SDS-PAGE gels, three distinct bands that appeared to specifically bind and copurify with the CpG-B ODNs were detected (Figure 5). These bands appeared only in lanes loaded with samples from cells treated with biotinylated ODNs. The intensity of their staining correlated with the concentration of the ODNs and most importantly, stimulation of the cells in the presence of an excess of unlabelled CpG-B ODNs inhibited their purification. These bands were isolated and subjected to matrix-assisted laser desorption/ionization-time-of-flight (MALDI-TOF) analysis, however, due to the extremely low protein amount, most of which is destroyed by the silver-staining, no proteins could be identified. Therefore, an alternative approach was undertaken in order to circumvent the need to isolate protein bands through SDS-PAGE/silver staining. To reduce the background, instead of boiling in the presence of LDS sample buffer, the proteins were released from the magnetic beads through incubation with either a combination of unlabelled CpG ODNs and biotin, or CpG ODNs only. The silver stained gels shown in Figure 5B demonstrate that, through this approach, the contamination of the samples with proteins that non-specifically attach to the streptavidin-conjugated magnetic beads has been significantly reduced. Using a combination of unlabelled ODNs and biotin seemed to be a more efficient recovery method for proteins that bind CpG-ODNs; however, there were still non-specific bands which most likely include biotin-binding proteins. Incubation of the beads with the unlabelled ODNs resulted in a very low protein recovery; however, due to the superior purity of the sample, an aliquot of it was used directly for tryptic digestion and LC MS-MS analysis as described in “Methods”. The proteins identified in this sample are listed in Table 1.

**Table 1:** CpG ODN-binding proteins identified through LC-MS/MS

**Protein ID**	**Pred. MW (kDa)**	**Identified peptide sequence**	**Score**	**Expect**
*NCOA5*; Nuclear receptor coactivator 5 (ACI33352.1)	64.84	DLGMVVDLIFLNTEVSLTQALEDVGR	128	1.7e-012
		TPFAIIITQQHQVHR	54	6.8e-005
		RYDTDRPVDCSVIVVNK	3	19
*hnRNPA0*; Heterogeneous nuclear ribonucleoprotein A0 (ACI32918.1)	31.5	LFVGGLNVDTDDDGLRK	60	4.3e-005
		REDAGKPEALAKV	10	11
*ACTB*; Actin, cytoplasmic 1 (ACI67042.1)	41.76	RVAPEEHPVLLTEAPLNPKA	35	0.0031

**Figure 6:**
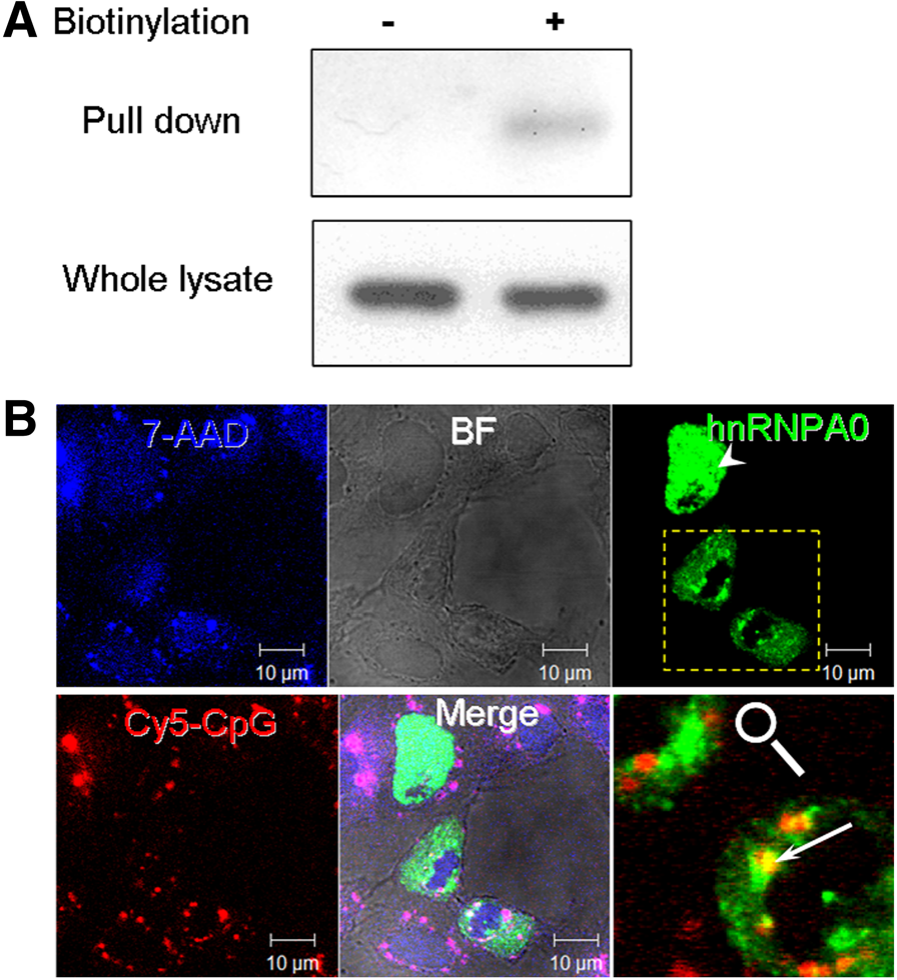
**Salmon hnRNPA0 binds CpG-B ODNs and colocalizes with CpG-B ODNs. A**, Western blot with mouse anti-hnRNPA0 antibody confirms that the salmon homolog expressed in mononuclear phagocytes interacts with CpG-B ODNs. The experiment was performed as in Figure 1A. **B**, Transgenic salmon hnRNPA0 is detected both in cell nuclei and cytoplasm and it colocalizes with fluorescent CpG-B. CHSE cells were transfected with a plasmid expressing an EGFP-hnRNPA0 fusion protein (green) and stimulated with Cy5-CpG-B as in Figure 2. The transgenic hnRNPA0 was observed mostly in cell nuclei (arrowhead); however, in cells which appear to be in a process of cytokinesis (boxed area), hnRNPA0 can be observed in cytoplasmic granules where it colocalizes with Cy5-CpGB (Arrow). The cell nuclei were counter-stained with 7-AAD (blue pseudocolor). Note that the emission, but not the excitation, spectrum of 7-AAD overlaps with that of Cy-5 and therefore, this channel detects both the genomic DNA and the endocytosed CpGs.

Of these, NCOA5 and hnRNPA0 contain RRMs domains which are known for their binding affinity for RNA and DNA and may, potentially, be involved in direct binding to the CpG-B ODNs in the current study. A third identified protein, actin-beta (ACTB), is a major component in the cell cytoskeleton. It is involved in multiple cellular functions and is known to interact with multiple targets. Therefore, its copurification with CpG-B ODNs may be mediated through other proteins that directly bind to the ODNs. Interestingly, hnRNP A0 protein has previously been shown to associate with CpG ODNs in pull-down experiments using a mammalian microglial cell line [23]. In the current study, using an antibody recognizing hnRNPA0 the interaction with the ODNs was confirmed. As seen in Figure 6A, hnRNPA0 specifically coprecipitates with CpG-B-ODNs.

It is well known that, like other members of its populous family, hnRNPA0 is located predominantly in the cell nucleus; however, as RNA-binding proteins some hnRNPs are shuttled between the nucleus and the cytoplasm and in the latter they may associate with RNA granules [24]. As shown in Figure 6B, overexpression of salmon hnRNPA0 results both in nuclear and cytoplasmic localization of the transgenic protein. Whereas most of the GFP-labelled fusion protein has nucleic distribution, in doublets, which appear to be in the process of, or have recently finished cytokinesis, hnRNPA0 is also seen in the cytoplasm. In some cells, cytoplasmic granules of the fusion protein also colocalize with endocytosed CpG-B ODNs thus indicating that the specific interaction detected with the pull-down experiments has the potential to occur in live cells and it is, probably, not merely an artefact of the experimental procedures.

## Discussion

The major goal of the current study has been to investigate the potential of SsTLR9 to serve as an immune receptor for CpG ODNs and to identify additional proteins involved in the recognition of these molecules in salmon innate immune cells. The presented results show that SsTLR9 does have the capacity to participate in the recognition of CpGs in Atlantic salmon mononuclear phagocytes as specific binding has been demonstrated. Unlike another study in which ODNs were added to cell lysates prior to coprecipitation [25], in the current research, the cells were first stimulated with the ligand, washed extensively and then lysed and processed for coprecipitation. This approach results in relatively poor protein recovery. However, it should be considered that, eukaryotic cells express large numbers of proteins with sequence-dependent and independent binding affinity to different types of nucleic acids. Therefore, the experimental approach where cell lysates are mixed with labelled nucleic acids could result in isolation of proteins that in live cells may not be able to interact with the immunostimulatory ligands due to spatial sequestration. Furthermore, the ligand-binding studies for TLRs can be additionally complicated by the specific conditions needed by some TLRs to assume efficient ligand-binding conformation. This is particularly important for endosomal TLRs, including TLR9, which has been shown to require proteolytic cleavage and low pH in order to be able to bind and activate immune response to foreign nucleic acids [26]. In agreement with this, in the current study adjusting the pH of the lysis buffer resulted in more efficient SsTLR9 recovery. In this case, the low pH most likely prevented the dissociation of the CpG that had already been associated with the receptor in the endosomes. While it is still possible that some of the coprecipitated SsTLR9 might have interacted with the ODN after the lysis, the immunostaining confirmed that SsTLR9 colocalizes with CpG-B ODNs in intracellular vesicles. The fact that most of the CpG-positive vesicles were also LysoSensor-positive indicates that these are maturing endolysosomes (pH < 5.2). These results are in agreement with previous studies with primary trout macrophages that have shown that the immunostimulatory capacity of CpGs depends on processes in which endosomal acidification is required [18].

Studies on mammalian TLR9 have indicated that binding to ODNs is not sequence-dependent, while the activation of the receptor relies on CpG-induced conformational changes [27]. Furthermore, it has been demonstrated that in order to assume efficient ligand-binding conformation, TLR9 needs to be processed by endosomal proteases [9,28]. This is considered to be a mechanism through which the cells control the signalling of TLR9 and other nucleic acid-binding TLRs and prevent their activation by endogenous ligands. In the current study, no evidence for proteolytic processing of SsTLR9 could be detected as only the predicted full-length molecule (~120 kDa) was specifically detected on the WB. The pull-down study also demonstrated that it is the full-length SsTLR9, rather than any proteolytically processed forms, that binds the ODNs. It light of the studies mentioned above, it might be questioned whether the SsTLR9 detected in the current study represents the functional receptor. Although the processed form of murine TLR9 had a higher potential to interact with DNA and signal the presence of CpGs, the full-length receptor also had some functional capacity. Furthermore, in another study, the authors showed that a chimeric TLR9/TLR4 receptor, which translocated to the cell membrane instead to the endolysosomal compartments, was still able to recognize DNA although it had not been proteolytically processed [29]. This study highlights the importance of the receptor localization for the control of the immune response to foreign and endogenous DNA molecules. Interestingly, the place of the proteolytic cleavage which is found between residues 441 and 470 of mouse TLR9 [9] lies within a phylogenetically poorly-conserved region. Sequence alignment of teleost and mammalian TLR9 sequences has shown that, in addition to the relatively low amino acid identity, in teleosts this region harbours a large insertion that is absent in the mammalian sequences [15]. Therefore it is possible that the proteolytic processing of TLR9 might not be present in teleosts. Despite these differences, the current study has demonstrated that the ODN-binding capacity of SsTLR9 is pH dependent indicating that the DNA-binding properties of SsTLR9 in salmon leukocytes might still be restricted to the endolysosomes,.

The translocation of TLR9 from the endoplasmic reticulum relies on its association with other molecules such as the polytopic membrane protein UNC93B1 [30]. In the absence of functional UNC93B1, TLR9 cannot be translocated to the endolysosomal compartments. Therefore, the difference between the intracellular distribution of endogenously expressed SsTLR9 and its overexpression in cell lines may be explained by the absence of the appropriate molecular mechanisms for redistribution of SsTLR9 in the cell lines. It is possible that CHSE and TO cells lack the machinery required for targeting the receptor to the endosomes. This may also explain why transgenic SsTLR9 did not augment the response to CpGs in CHSE cells. Nevertheless, the SsTLR9 overexpression spontaneously activated the activity of promoters that contain ISRE elements. This indicates that the cloned molecule is functional and highlights the potential of the receptor to mediate IFN upregulation and induction of antiviral genes in response to pathogens.

As revealed by the current study, the CHSE cells do not express detectable levels of endogenous TLR9 protein and they are not able to relocate the transgenic SsTLR9 to compartments where it may be activated by CpG ODNs. However, these cells can efficiently endocytose and accumulate ODNs within late endosomes and they do respond to stimulation with different immunostimulatory nucleotides as demonstrated by the RGAs. This suggests that CHSE cells use alternative receptors that allow them to respond to ODNs by activating promoters of IFN-responsive genes.

As TLR9 is an intracellular receptor that is not involved in DNA uptake, it is obvious that the uptake and the distribution of exogenous DNA by cells rely on other molecules. The endocytosis of CpG ODNs by immune cells has been suggested to be dependent on receptor-mediated endocytosis, more specifically on the function of scavenger receptors [31]. However, the exact mechanisms that regulate the distribution of different types of CpG ODNs within specific endosomal compartments are still obscure. Studies have also suggested that, in addition to TLR9, there are other molecules that mediate the signalling properties of immunomodulatory DNA sequences [31,32]. The isolation of proteins that specifically bind CpG ODNs and that could serve as mediators of the biological activity of CpG ODNs in primary cells, requires development of techniques for both efficient and specific protein purification. The results presented in the current paper suggest that hnRNPA0 and NCOA5 may, potentially, be involved in the CpG response. Unlike ACTB which probably copurifies with CpG ODNs through indirect binding, both hnRNPA0 and NCOA5 possess RRM domains which have binding affinity for both RNA and single-stranded DNA suggesting direct interaction with the ODNs. Of these molecules, hnRNPA0 is particularly interesting since it has been previously identified as a protein which specifically binds to CpG ODNs in rat gliobastoma cells [25]. In this study the authors also identified a number of other ODN binding proteins; however, the experimental approach chosen by these researchers (i.e. adding the ODNs to the cell lysates rather than stimulating viable cells) allows for the isolation of proteins that in viable cells may not have access to the endocytosed CpGs.

The hnRNPA0 belongs to a protein family whose members have predominantly nuclear distribution and, therefore, it is questionable whether the observed interaction may be an artefact issuing from the binding of the protein to CpG ODNs released by endosomes after the cell lysis. However, the experiments with transgenic salmon hnRNPA0 presented here demonstrate that in dividing CHSE cells this molecule is found mostly in the cytoplasm. In the current paper, it is also shown that the hnRNPA0-GFP fusion protein colocalizes with CpG ODNs in granular structures within the cytoplasm of CHSE cells indicating that it is likely that the observed interaction has occurred in the live cells prior to, rather than after the cell lysis and that this binding might have biological significance..

The functions of mammalian hnRNPA0 are still poorly characterized. Since the general functions of the members of the hnRNP family are, posttranscriptional RNA processing and transport [33], it is not very likely that hnRNPA0 may be directly involved in the intracellular signalling events triggered by CpGs. Interestingly, it has been suggested that, in mouse, this protein might be implicated in the regulation of the immune response to LPS stimulation [34]. More specifically, upon LPS stimulation of mouse macrophages, this protein binds to AU-rich elements (AREs) of Cyclooxygenase-2 (COX-2), TNF-α and macrophage inflammatory protein-2 mRNAs suggesting a role in the regulation of their stability and the cytokine production. Currently, there is no functional data linking the capacity of hnRNPA0 to bind CpG ODNs with its potential to regulate cytokine stability. In this regard; further studies in this direction would be relevant.

## Conclusions

The current study has indicated that SsTLR9 has the capacity to bind PS-modified oligonucleotides through a sequence-independent but a pH-dependent mechanism. The potential of this receptor to activate IFN responses is highlighted by the presented functional studies showing its capacity to activate ISRE-containing promoters. The data further indicate that the redistribution of this receptor and its colocalization with CpG ODNs within endosomal compartments is cell type-dependent. Finally, the study identifies the nucleotide-binding proteins hnRNPA0 and NCOA5 as CpG ODN-binding proteins. Further studies are needed to investigate the sequence specificity of these interactions and to investigate whether these proteins might have a role in modulating the cellular response to foreign DNA molecules.

## Methods

### Fish

Atlantic salmon (*Salmo salar*) strain Aquagen standard (Aquagen, Kyrksæterøra, Norway), of 500 g, were obtained from the Tromsø Aquaculture Research Station (Tromsø, Norway). The fish were kept at about 10°C in tanks supplied with running filtered water and were fed on commercial, dry food (Skretting, Stavanger, Norway). All experiments were performed according to the guidelines from the national committee for animal experimentation (Forsøksdyrutvalget, Norway).

### Isolation and stimulation of primary leukocytes from Atlantic salmon

Head kidney (HK) leukocytes were isolated as described by Strandskog et al. [19]. The cells were seeded in 60 mm cell culture dishes at a density of 2.1 × 10^7 cells per dish. After 24 h of incubation, the cells were washed with fresh L15 medium and incubated for another 24 h in L15 supplemented with 5% FBS and antibiotics prior to treatment. To up-regulate SsTLR9 protein, the cells were treated with 50 U/ml of recombinant rainbow trout IFN-γ [20] for 48 hours prior to stimulation. The stimulations (2 hours) were performed with specified concentrations of CpG-B (2006T: TCGTCGTTTTGTCGTTTTGTCGTT) or inverted, non-stimulatory CpG-B ODN (2007T: TGCTGCTTTTGTGCTTTTGTGCTT) (Eurogentec). All of the phosphodiester links were substituted with PS bonds. The ODNs were biotinylated a using PHOTOPROBE® (Long Arm) Biotin kit (Vector labs) according to the manufacturer’s instructions. Briefly, 400 μM ODNs dissolved in TE buffer (pH 8) were mixed with an equal volume of PHOTOPROBE® (LA) Biotin, citrate salt (FW = 838.9) in a PCR tube. The mixture was subjected to thermal coupling by heating to 95°C for 30 min. The reactions were purified to remove unlabelled bioting with Zeba™ Spin Desalting Columns, 7 K MWCO (Pierce). The cells were stimulated with 2 μM biotinylated or unlabelled ODNs unless otherwise indicated in the figures.

All experiments were performed according to the guidelines from the national committee for animal experimentation (Forsøksdyrutvalget, Norway).

### Pull-down procedures

Following the stimulations the cells were washed 3x with PBS, to remove non-incorporated ODNs, and lysed in 500 μl of lysis buffer (50 mM Tris-HCl, pH 7.5, 150 mM NaCl, 2 mM EDTA, 1 mM EGTA, 1% Triton X-100) supplemented with a protease inhibitor mixture (Complete EDTA-free; Roche Applied Science). To analyse the TLR9 binding efficiency under different pH conditions, the acidity of the lysis buffer (original pH 7.5) was adjusted using 3 M HCl. The cell lysates were cleared by centrifugation for 15 min at 15,000 × *g*. and 50 μl of Streptavidin-coupled Magnetic Beads (Dynabeads®, Dynal) were added to each sample. The Magnetic Beads were washed in lysis buffer prior to addition. The samples were rotated for 45 min at 4°C and washed 5x with lysis buffer using a microcentrifuge tube magnetic stand. For the Western blot analysis, the proteins were eluted by heating the beads (70°C for 10 min) in LDS sample buffer (Life Technologies). The samples used for mass spectrometry were eluted using milder elution conditions - the beads were incubated for 20 min in 50 μl of lysis buffer supplemented with 0.5 μg/ml of biotin and 2 μM ODNs or 2 μM ODNs only.

### Western blot (WB)

The protein samples prepared in LDS buffer were analysed as previously described [35] using NuPAGE Novex Bis-Tris 4–12% gels (Life Technologies). The SsTLR9 antibody has been previously characterized [15] and was used at 1:1000-fold dilution. The anti-mouse hnRNP antibody [34] was kindly provided by Dr Simon Rousseau, University of Dundee and was used at 1:500 dilution. The membranes probed with the SsTLR9 antibody were developed with the SuperSignal West Femto ultra-sensitive substrate whereas hnRNPA0 signal was developed with the less-sensitive SuperSignal West Pico substrate (Pierce). The membranes were exposed to Lumi Film Chemiluminescent Detection film (Roche).

### Immunocytochemistry and confocal microscopy

Primary mononuclear phagocytes, Chinook salmon embryo CHSE-214 cells [36] and salmon head kidney-derived TO cells [37] were incubated on coverslips and processed as previously described [38]. The SsTLR9 antibody was applied using a 1:1000-fold dilution. To visualise endolysosomes in live cells, CHSE cells grown in Lab-Tek™ Chambered Coverglass slides (Nunc) were incubated with 1 μM LysoSensor™ Green DND-189 (Life technologies) for 30 min, washed and subjected directly or following the indicated stimulations with CpG-B-Cy5 (Eurogenetech) to confocal microscopy. The transfections of CHSE and TO cells were performed using FuGeneHD (Roche) as previously described [35], The expression constructs include pDEST12.2 (Invitrogen), pDESTEGFP C1 [35] and pDEST12.2-SsTLR9 [39]. SYTOX^®^ Green and 7-aminoactinomycin D (7-AAD), which were used to stain cell nuclei, were purchased from Life Technologies. The images were taken using an Axiovert 200 microscope equipped with an LSM510-META confocal module (Zeiss).

### Reporter gene assays (RGA)

The RGAs were performed as previously described [35]. The reporter that contains the promoter region I of the Atlantic salmon IFNa1 [21] was provided by Prof. Børre Robertsen (University of Tromsø, Norway). The rainbow trout Mx1 promoter construct was described earlier [22]. The ISRE reporter was purchased from SABiosciences. The Poly(I:C) was purchased from Invivogen. PS-modified CpG-B and CpG-A (ODN 2216TGGGGGACGATCGTCGGGGGG) were obtained from Eurogenetech. The firefly luciferase values were normalized using the Renilla luciferase levels, and the values were presented as relative luciferase units (RLU).

### Mass spectrometry (MS)

To analyse the efficiency of the pull-down and the purity of the coprecipitation, the samples were subjected to SDS-PAGE, as described above, and silver staining using the SilverQuest™ Silver Staining Kit (Life Technologies). Samples which had lowest background were further analysed with MS to identify potential CpG ODN-binding proteins. The MS analysis was performed at the Proteomic Unit at University of Bergen (PROBE). The protocol included: **1. Reduction and alkylation;** Possible -S-S- bridges were reduced with DiThioTreitol from Amersham Biosciences, #171318-02) followed by alkylation with iodoacetamide (Sigma Aldrich, I-6125), to prevent oxidation and formation of new -S-S- bridges. **2. Digestion (peptide fingerprint);** Porcine Trypsin from Promega (#V 511A) was used to digest the samples (16 hours at 37°C). The protease activity was stopped by acidification using 1% TFA (Trifluoroacetic acid). **3. Filtering/desalting of samples;** An nC18 StageTip (Stop and go extraction Tip) column (Rappsilber, Ishihama et al. 2003) was used. The bound peptides were eluted by passing 10 μl 70% acetonirile/0.1% formic acid through the column. **4. Nano-LC** was performed with Ultimate 3000 nano-LC (Dionex Corporation, USA) with a nC18 enrichment column (C18 Pepmap 100 from Dionex, 5 μm particle size, 100 Å pore size, 300 μm i.d. × 5 mm) and an nC18 analytical column (C18 Pepmap 100 from Dionex; 3 μm particle size, 100 Å pore size, 75 μm × 150 mm). **5. Mass spectrometry;** The eluted peptides from the nano-LC were ionized (ESI, ElectroSpray Ionization) and analyzed with a QToF Global (Quadrupole Time of Flight from Waters, USA) using automatic MS to MSMS switching (DDA, Data Dependent Analysis). **6. MS/MS search;** The mass spectra of fragmented peptides were smoothed (Savitzky Golay), centered, and combined in a merge file. This merge file was then used to search the NCBInr protein database. Mascot Daemon (http://www.matrixscience.com/daemon.html) was used to search the obtained MSMS spectra in a subset of the NCBInr database consisting of protein sequences from Class Actinopterygii (ray-finned fishes).

### Data analysis

The RGA data was analysed with two-way ANOVA, followed by Bonferroni post-tests. Statistical analyses were performed using the GraphPad Prism6 software. The value of p < 0.05 was considered to be significant.

## Abbreviations

TLR: Toll-like receptor;ODNs: Oligodeoxynucleotides;MyD88: Myeloid differentiation primary response gene 88;NFkB: Nuclear factor kappa-B;IRF7: Interferon-regulatory factor 7;IFN: Interferon;TNFα: Tumor necrosis factor-alpha;PS: Phosphorothioate;ISRE: IFN-stimulated response element;RRM: RNA recognition motifs;hnRNPA0: Heterogeneous nuclear ribonucleoprotein A0;NCOA5: Nuclear receptor coactivator 5;WB: Western blot;CHSE: Chinook salmon embryo;RGAs: Reporter gene assays;CMV: Cytomegalovirus;EV: Empty vector;EGFP: Enhanced green fluorescent protein;MALDI-TOF: Matrix-assisted laser desorption/ionization-time-of-flight;MS: Mass spectrometry;ACTB: Actin-beta;AREs: AU-rich elements;COX-2: Cyclooxygenase-2;HK: Head kidney;SDS-PAGE: Sodium dodecyl sulfate polyacrylamide gel electrophoresis;PCR: Polymerase chain reaction;LC: Liquid chromatography;PBS: Phosphate-buffered saline

## Competing interests

The authors declare that they have no competing interests.

## Authors’ contributions

DBI carried out all the experiments for this study, participated in its design and data-analyses and drafted the manuscript. IS provided the SsTLR9 constructs and initiated some of the reporter gene assay studies. JBJ initiated and coordinated this study and edited the manuscript. All authors have read and approved the final manuscript.
